# Attempts to evaluate locus suicide recombination and its potential role in B cell negative selection in the mouse

**DOI:** 10.3389/fimmu.2023.1155906

**Published:** 2023-06-09

**Authors:** Nicolas Denis-Lagache, Christelle Oblet, Tiffany Marchiol, Audrey Baylet, Ophélie Têteau, Iman Dalloul, Zeinab Dalloul, Lina Zawil, Ophélie Dézé, Jeanne Cook-Moreau, Alexis Saintamand, Hend Boutouil, Ahmed Amine Khamlichi, Claire Carrion, Sophie Péron, Sandrine Le Noir, Brice Laffleur, Michel Cogné

**Affiliations:** ^1^ Limoges University, Centre National de la Recherche Scientifique (CNRS), Limoges, France; ^2^ Rennes University, Inserm, Rennes, France; ^3^ Institut de Pharmacologie et de Biologie Structurale, Centre National de la Recherche Scientifique (CNRS), Toulouse University, Toulouse, France

**Keywords:** antibody, immunoglobulin, lymphocyte, enhancer, recombination, mouse model

## Abstract

**Introduction:**

In mature B cells, activation-induced deaminase reshapes Ig genes through somatic hypermutation and class switch recombination of the Ig heavy chain (*IgH*) locus under control of its 3’ *cis*-regulatory region (*3’RR*). The *3’RR* is itself transcribed and can undergo “locus suicide recombination” (LSR), then deleting the constant gene cluster and terminating *IgH* expression. The relative contribution of LSR to B cell negative selection remains to be determined.

**Methods:**

Here, we set up a knock-in mouse reporter model for LSR events with the aim to get clearer insights into the circumstances triggering LSR. In order to explore the consequences of LSR defects, we reciprocally explored the presence of autoantibodies in various mutant mouse lines in which LSR was perturbed by the lack of Sµ or of the *3’RR*.

**Results:**

Evaluation of LSR events in a dedicated reporter mouse model showed their occurrence in various conditions of B cell activation, notably in antigen-experienced B cells Studies of mice with LSR defects evidenced increased amounts of self-reactive antibodies.

**Discussion:**

While the activation pathways associated with LSR are diverse, *in vivo* as well as *in vitro*, this study suggests that LSR may contribute to the elimination of self-reactive B cells.

## Introduction

Antigen (Ag)-specific antibody (Ab) responses rely on cells harboring B cell receptors (BCRs) optimally selected within germinal centers (GCs). Activation-induced deaminase (AID)-dependent somatic hypermutation (SHM) carves high affinity immunoglobulin (Ig) variable (V) domains in cells which then more efficiently capture Ags and undergo the best cognate interactions with T follicular helper cells. In parallel, class switch recombination (CSR) of the Ig heavy (*IgH*) chain locus replaces expression of the constant (C)µ gene with a downstream C gene by joining the repetitive switch (S) regions of C genes. After successful SHM and/or CSR, cells with reshaped BCRs either differentiate into plasma cells and secrete high affinity Abs or eventually survive as memory cells. Positive selection of such cells inherently requires simultaneous elimination of competitors. Terminating immune responses also later implies contraction of immune cell populations through programmed cell death pathways. GC B cells face multiple pro-apoptotic risks. They strongly express proapoptotic proteins such as BAX, BAD, BID and FAS receptor (while down-regulating anti-apoptotic proteins BCL2 and BCL-XL). Their active metabolism yields abundant reactive oxygen species and they accumulate DNA lesions including mismatches, apurinic/apyrimidic (AP) DNA and single- or double-strand breaks. Meanwhile, the BCL6-driven program protects activated cells from death by down-regulating P53, P21, CHK1 and ATR; expression of the AP endonuclease APE2 and the polymerase Rev7 also protects cells from toxic AP-lesions and from the DNA damage response (1, 2).

Altogether, specific immune responses need to restrict survival signals to *bona fide* Ag-specific cells while dampening activation of polyreactive or passenger B cells transiting through lymphoid tissues and undergoing sub-optimal stimulation. Fine tuning of the pro-survival *vs* pro-apoptotic pathways is thus a major issue in physiology, in order to prevent lymphomagenesis or deregulated expansion of inappropriate B cells.

While immature and transitional cells can readily die upon activation, mature B cells are more resistant to apoptosis (3). Besides the successful outcomes of activation, death however remains a frequent fate for GC and recent post-GC B cells (4, 5). This combines signals such as those provided by Fas or CD22 (6, 7), death related to DNA lesions (1, 8), *death-by-neglect* for cells lacking BCR signals (9, 10) and *activation-induced cell death* (AICD). B cell AICD involves factors such as EndoU or the transcription factor Fra1 (11, 12) and can notably follow excessive BCR/TLR signals (13), but also BCR cross-linking in the absence of second signals from T-cells or TLRs (14, 15).

Beyond the tonic signal provided by BCR expression, BCR cross-linking triggers activation, which associates with AID-dependent SHM and CSR remodeling of the *IgH* locus under control of its 3’ regulatory region (*3’RR*) (16–20). Accessibility of S regions to CSR relies on promoters responsive to cytokines released after B-T interactions. By contrast, the *3’RR* controlling these promoters, does not itself bind cytokine-dependent transcription factors but rather those typical of B cell commitment and activation (such as PAX5, PU1, OCT, NF-κB, ETS1, AP1…) (21, 22). Local chromatin remodeling, physical accessibility and transcription of the *3’RR*, yielding enhancer (e)RNA, are thus mostly modulated by B cell activation (23, 24).

Fate of such activated B cells differentially relies on AID-mediated effects. Besides affinity maturation, unfavorable V region SHM or *IgH* locus recombination can promote apoptosis by altering Ag recognition or Ig expression (4, 24). We have previously discovered a CSR-like, AID-mediated event joining an S region to the 3’RR, dubbed locus suicide recombination (LSR), that leads to the loss of the whole constant region and shut-down of Ig expression (24, 25). To what extent lost BCR expression contributes to B cell homoeostasis in the context of LSR remains to be clarified. Various non-mutually exclusive hypotheses can be put forward for the role of LSR including elimination of bystander cells with non-cognate BCR, of low-affinity cells receiving incomplete T-cell help, and/or of harmful cells having acquired self-binding affinity for self Ags. Since switched Abs are the most pro-inflammatory, means for limiting CSR of self-reactive cells could be of interest for the specificity and safety of immune responses.

As a first attempt to explore the contribution of LSR to negative selection and B cell AICD pathways, we designed a LSR reporter model in which we modified the murine *IgH* locus by inserting a V-less Ig human Cµ (hCµ) gene downstream of the *3’RR*. The *3’RR* enhancers (hs3a, 1-2, 3b and 4) are interspersed in all mammalian species with highly repetitive “like-Switch” (LS) stretches (with eleven LS regions in the mouse). LS1 precedes hs3a; LS2 to LS7 lie in the palindrome flanking hs1-2; LS8-9 follow hs3b and LS10-11 follow hs4 (24). Having previously noticed that LS repeats functionally mimic S regions by recruiting AID (and then undergoing LSR), we knocked-in the reporter human Cµ downstream of the most downstream LS region, thus conditioning its inducible expression on the occurrence of LSR junctions, then able to yield primary transcripts made up of mouse VDJ and hCµ. Such transcripts should be spliced into mature VDJ-hCµ transcripts, ultimately leading to chimeric mouse-human µ H chain. This reporter model confirmed that a Cµ gene inserted downstream of potential LSR breaks could be expressed at this position after B cell activation.

Additionally, we evaluated the effect of an LSR defect in mouse models invalidated for *Sµ* or *3’RR*, sequences which normally support LSR recombination, and observed a significantly increased occurrence of self-binding Abs in such models, suggesting a contribution of LSR to B cell negative selection.

## Materials and methods

### Mice

The procedures for mouse studies have been reviewed and approved by the Ministère de l’Education Nationale de l’Enseignement Supérieur et Recherche (agreement APAFIS# 16274-2018072514307380v9). Mice were bred in a specific and opportunistic pathogen free (SOPF) core facility. In addition to wild-type (*wt*) controls, samples used in this study originated from previously described transgenic mice including mice with a complete deletion of *Sµ* (26), *3’RR*-deficient animals (*3’RR*KO) (19), c*3’RR* mice with a partial 3’RR deletion (27), µϵKI mice expressing human IgM heavy chains (28), together with Rag-deficient mice (negative controls lacking circulating Ig), and MRL/lpr mice (positive controls for circulating autoantibodies).

### Generation of reporter LSRµKI mice

**Figure 1:**
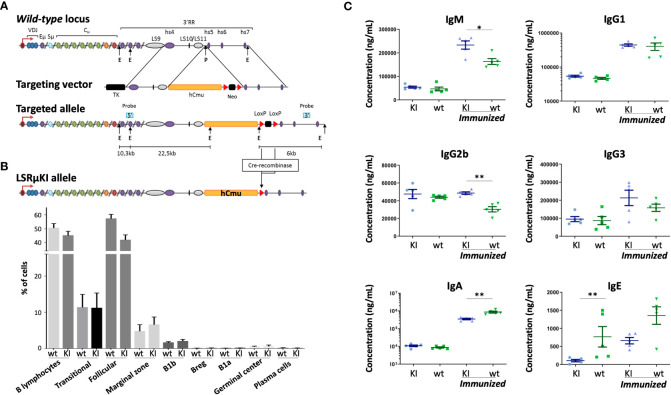
Characterization of LSRµKI mice. **(A)** Map of the murine IgH locus and the targeting vector used for insertion of the hCµ gene in-between the hs4 enhancer and the hs5 insulating element, downstream of LS10-11 repeats (TK, thymidine kinase gene; E, EcoRI; P, PmlI) (not to scale). **(B)** Minimal impact of hCµ knock-in on peripheral B cell compartments as shown by cell cytometry analysis of splenocytes. **(C)** Secretion of mouse Ig in LSRµKI mice in blood monitored by ELISA. (n = 5 to 6 mice, Mann and Whitney non-parametric two-tailed test; *P < 0.05; **P < 0.01).

A complete genomic DNA fragment including the same full-length human Cµ gene previously expressed at high levels in µϵKI mice (28), with the complete CH1,2,3,4, M1 and M2 exons and intervening introns, followed by a floxed neomycin resistance gene (*neo* ^R^) was introduced in between 5’ and 3’ arms for homologous recombination and insertion downstream of the hs4 core enhancer and the LS11 like-switch repeats (24), and upstream of the elements defined as the 3’ boundary of the *IgH* locus, *i.e.* the hs5, hs6 and hs7 elements and associated CTCF binding sites (29) (**Figure 1A**). At the 5’ end of the targeting construct, a phosphoglucokinase promoter-Herpes Simplex Virus thymidine kinase (TK) gene was included in order to negatively select against random integration. Cells of the embryonic stem (ES) cell line E14 were transfected with linearized vector by electroporation and selected using 300 μg/ml geneticin and 2 μg/ml ganciclovir. Southern blot analyses with probes hybridizing 5’ and 3’ of the construct and specific PCR experiments identified recombinants. ES clones showing homologous recombination were injected into C57Bl/6 blastocysts, and the resulting chimeras were mated with C57Bl/6 animals. Germline transmission in heterozygous mutant mice was checked by Southern blot. Mutant mice were further mated with *cre* recombinase-expressing transgenic mice and progeny were checked for *cre*-mediated deletion of the *neo* ^R^ gene. Mice were further genotyped with a triple PCR assay to simultaneously evaluate the presence of the hCµ gene, absence of the *neo* ^R^ gene and the homogeneous loss of the WT site targeted downstream of the *3’RR*.

### Blood sampling in mice

Blood samples were recovered from transgenic mice and *wt* controls with heparinised needles. Plasma samples were recovered by centrifugation and stored at -20°C until used. Immunization with sheep red blood cells (SRBC, 200 µL per immunization) was done by intraperitoneal injection. Blood samples were taken at day 0 and day 8 after injection and assayed for human IgM by ELISA.

### Spleen cell cultures for Ig determinations

Spleens were harvested at day 8 post-immunization. Splenocytes were collected, red blood cells were lysed and cells were CD43-depleted using CD43 microbeads (Miltenyi Biotec). Single-cell suspensions of CD43^-^ B splenocytes from *wt*, and LSRµKI mice were cultured 3 days at 1x10^6^ cells/ml in RPMI 1640 with 10% fetal calf serum, 5μg/ml LPS with or without, 20ng/ml IL-4, 2ng/ml TGFβ and 2 ng/ml INFγ (PeproTech, Rocky Hill, NJ), anti-CD40 antibody (2.5μg/ml) (R&D systems) and anti-κ light chain antibody (2.5µg/ml, Southern Biotechnology) for BCR cross-linking. Culture samples were harvested at day 4 for RNA extraction. Culture supernatants were then recovered and stored at -20°C until used.

### Immunization and ELISA assays

For immunization experiments, batches of 8-week-old mice were used (6 mice per genotype). The first immunization was performed with antigen (50 μg NP-Ficoll in 50% incomplete Freund adjuvant or 100µg NP-KLH in 50% complete Freund adjuvant per animal) and a second immunization was realized 30 days later with the same amount of antigen in 50% incomplete Freund adjuvant. Immunized mice were eye-bled at various intervals during the immunization protocol and plasma was analyzed for the presence of antigen-specific IgM, IgG_1_, IgG_2a_ and IgG_2b_ by ELISA as previously described (27).

Assays for specific IgM, IgG_1_, IgG_2a_ and IgG_2b_ were performed in plates coated with 10µg/ml antigen in 0.05 M Na_2_-CO_3_ buffer. After washing, a blocking step was performed with 3% bovine serum albumin (BSA) – PBS. For analyses, serum or plasma were diluted into successive wells in 1% BSA - PBS and incubated 2 hours at 37°C. The positive control consisted of a pool of plasma from immunized *wt* mice (the same control plasma was used in all ELISA assays). After washing, 100 μl/well of appropriate conjugated antibodies were added and adsorbed during 1 hour at 37°C. Alkaline phosphatase (AP)-conjugated goat antisera specific for human IgM, mouse IgM, IgG_1_, IgG_2a_ and IgG_2b_ (Southern Biotechnologies) were used at 1 μg/ml. After washing, AP activity was assayed using AP substrate and enzymatic reactions were stopped with 3 M NaOH. Optical density was measured at 405 nm. Ovalbumin-specific antibodies were quantified in arbitrary units by comparing diluted plasma with the titration curve obtained on the same multiwell plate.

Plasma from LSRµKI and *wt* mice was analyzed for the presence of various mouse Ig classes (IgM, IgG_1_, IgG_2b_, IgG_2b_, IgG_3_, IgA) and for human IgM by ELISA. Assays were performed in polycarbonate 96 multiwell plates, coated overnight at 4°C (100 µl/well) with suitable capture antibodies diluted in 0.05 M Na_2_CO_3_ buffer. After washing, a blocking step was performed and 50 µl plasma (first diluted to 1:50), supernatants or isotypic standard Ig were diluted into successive wells in 1% BSA/PBS and incubated for 2 hr at 37°C. After washing, appropriate AP-conjugated goat antisera specific for human IgM or mouse Ig classes (Southern Biotechnologies) were added and adsorbed during 1 hr at 37°C. After washing, AP activity was assayed on AP substrate, and blocked by 3 M NaOH. Optic density was then measured at 405 nm.

### Cell cytometry

For cytometry analysis, single cell suspensions of splenocytes from 4- to 8-week old mice, were stained with the following monoclonal antibodies conjugated to either fluorescein isothiocyanate (FITC), phycoerythrin (PE), allophycocyanin (APC), phycoerythrin-cyanin 7 (PECY7) or phycoerythrin-cyanin 5 (PC5): anti-CD5 (53-7.3), anti-CD19 (6D5), anti-CD23 (B3B4), anti-CD21/CD35 (7G6), anti-B220 (RA3-6B2) (all from BD Pharmingen, Southern Biotech or e-bioscience).

### Real time quantitative PCR

Four-day *in vitro* stimulated splenocytes were harvested and RNA was extracted and reverse transcribed using the High-Capacity cDNA Archive Kit (Applied Biosystems) before being subjected to quantitative PCR (qPCR) to evaluate functional JH3-hCµ spliced transcripts. Quantitative PCR was performed using SYBR^®^ Green (Takara). Data for JH3-hCµ transcripts were normalized according to the parallel evaluation of β-actin transcripts.

### Repertoire analysis using RACE-PCR and next-generation sequencing

Total RNA was extracted using TRIzol reagent (ThermoFisher) from 5x10^6^ CD43- spleen B cells from 2 groups of 5 LSRµKI and 5 *wt* mice. For each sample, 500 ng total RNA was reverse-transcribed to cDNA by a 5’RACE PCR using ProtoScript II (New England Biolabs) and a cap race primer associated with consensus reverse primers for each mouse *IgH* Cμ, Cγ and Cα CH1 region or the human Cµ CH1 (as detailed in **Supplementary Table 1**). To prepare libraries, 5 µl cDNA were amplified with Taq Phusion (New England Biolabs), using a universal forward primer and a nested reverse primer selected within the CH1 exon of the Cμ, Cγ and Cα genes. This amplified region corresponded to the 5’ part of *IgH* transcripts; PCR products were purified using (0.6 x reaction volume) AMPure XP beads (Beckman Coulter), and resuspended in 20µl NEB elution buffer (Qiagen). Thereafter, 150ng amplified product was barcoded and tagged with sequencing adapters through 12 cycles of primer extension with Taq Phusion (30 seconds at 98°C, 30 seconds at 65°C, 30 seconds at 72) with a final elongation 5 minutes at 72°C. Tagged PCR products were purified using (0.6 x reaction volume) AMPure XP beads and mixed with a stoichiometric ratio. Resulting libraries were gel purified (QIAGEN) before sequencing. Sequencing adaptor sequences and tagging PCR steps were as previously reported for sequencing either with the Titanium emPCR Kit (Lib-A) on a 454 GS Junior instrument (Roche) or the MiSeq Reagent Kit v3 (600 cycles) with a MiSeq apparatus (Illumina) (30, 31). Ig classes and subclasses were identified by matching the included CH1 part to the relevant C gene. VDJ sequences sorted according to the associated C gene were then analyzed using high-VQUEST software (IMGT) (32). Redundant sequences were excluded in order to evaluate the global repertoire of clonotypes and the mean somatic hypermutation level.

### Data availability

*RepSeq* data have been deposited at GEO repository (accession number GSE233503) in order to be publicly available as of the date of publication. DNA repertoire reported in this paper will be shared by the lead contact upon request. Any additional information required to reanalyze the data reported in this paper is available from the lead contact upon request.

### Evaluation of self-reactive Abs

Plasma from mice carrying alterations of the 3′RR or deletion of *Sµ* were compared to plasma from *wt* animals. Plasma from Rag-deficient mice were used as negative controls and plasma from MRL/lpr mice as positive autoimmune controls. Since some evaluated mice carried CSR anomalies and the assays focused on IgM, all plasma assayed were first evaluated for their total IgM level and then diluted to a standardized total IgM level of 20-µg/mL (except the Rag-deficient serum which was diluted to 1:10).

Evaluation of Abs binding tissue antigens was done by ELISA assays in 96 multi-well plates. Plates were thus coated overnight at 4°C either with 10 μg/mL rabbit IgG (Dako, X0903) or with 100 μL kidney cell lysate (10-μg/mL, diluted in Na_2_CO_3_/NaHCO_3_ buffer). After removing coating solution, a blocking step was performed for 1 hour with 3% BSA- PBS at 37°C. After washing (0.1% Tween20 - PBS), plasma normalized according to their total IgM content were diluted in 0.3% BSA - PBS and incubated in ELISA plates for 2 hours at 37°C. After washing, secondary anti-mouse-IgM–alkaline phosphatase (AP)-conjugated antibodies (Southern Biotech, #1021-04) were added and incubated for 1 hour at 37°C. After final washing, ELISA were revealed with SigmaFAST™ p-Nitrophenyl phosphate (p-Npp) substrate (Sigma-Aldrich #N2770) and optical density was read at 405 nm.

ELISA were also carried out for quantifying rheumatoid factors (*i.e.* IgM anti IgG), by evaluating the binding of circulating Abs against rabbit IgG, as done for clinical assays in patients. To this goal, ELISA plates were treated as mentioned above, except that plates were initially coated with rabbit IgG. For all ELISA assays, data were expressed as means ± SEM of the indicated number (n) of values, as calculated using Prism software (GraphPad Software, La Jolla, CA). Significance was calculated with a nonparametric Mann-Whitney *U* test.

To detect Abs reactive to intracellular Ags, indirect immunofluorescence assays (IFAs) were adapted from clinical assays and performed according to the manufacturer’s instructions. In brief, HEp-2 cell coated slides (Kallestad™ HEp-2, Bio-Rad) were incubated at 37°C for 30min with sera adjusted to 20 µg/ml IgM. Slides were washed in PBS and incubated with Alexa Fluor^®^ 488 anti-mouse IgM (Life, A21042). Fluorescence was visualized on a Nikon Eclipse Ni-E microscope. Serum from MRL/lpr auto-immune mice was used as a positive control and RAG-deficient serum diluted to 1:10 was used as negative control. Images were analyzed with ImageJ software (NIH), converting the single channel color image to a binary image, segmented into features of interest. Areas corresponding to fluorescence signals were measured. Results are expressed as arbitrary units (measured fluorescence area of sample divided by measured fluorescence area of PBS). Data, expressed as means ± SEMs of the indicated number of values, were analyzed using Prism software (GraphPad Software, La Jolla, CA). Significance was calculated with a nonparametric Mann-Whitney test using Prism software.

## Results

### Peripheral B cells normally appear in LSRµKI mice

Since the mouse *3’RR* includes several stretches of repetitive like-switch (LS) sequences, we chose to insert a reporter Cµ gene between the most 3’ LS region and the insulating elements previously shown to act as the 3’ boundary and 3’ anchor of the *IgH* locus (29, 33) (**Figure 1A**). ES cells were transfected, selected using neomycin, screened and validated for efficient targeting before generating the homozygous LSRµKI mice (**Figures S1A, B**) used for this study. Peripheral B cells differentiated and their analysis among splenocytes by flow cytometry did not significantly differ from their wild-type counterparts in terms of global number, amount of transitional B cells, and distribution into follicular *vs* marginal zone B cells (**Figures 1B**; **S1C**), as well as early B cell development was not affected in LSRµKI mice (**Figure S1D**). Thus, the ectopic hCµ insertion downstream of the *3’RR* had no major impact on B cell development. Levels of circulating Ig by contrast suggested discrete alterations of the class switching process (**Figure 1C**). In basal SOPF conditions and without any immunization, LSRµKI mice in fact barely differed from WT animals, except for a lower IgE level (**Figure 1C**). Additional differences appeared in immunized mutant animals with IgA production being lower than in *wt* mice, while IgG1 and IgG3 showed no significant variation and endogenous mouse IgM and IgG2b levels were higher than in *wt* controls (**Figure 1C**).

**Figure 2:**
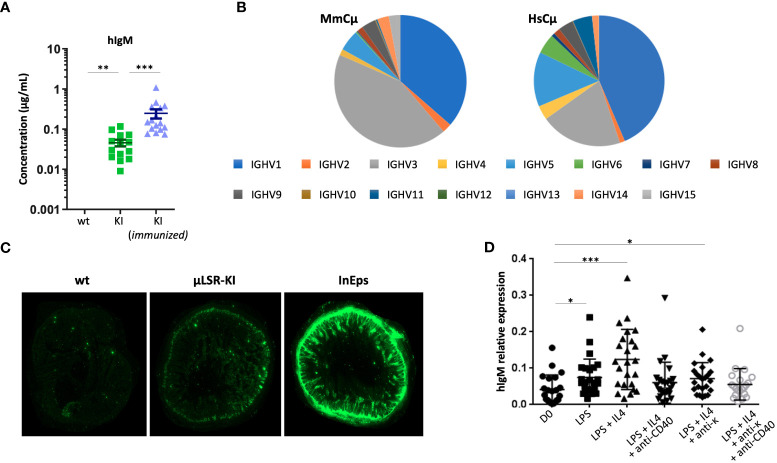
Expression of human *IgH* µ chain in LSRµKI mice. **(A)** ELISA of circulating human IgM in blood from naive or SRBC-immunized LSRµKI mice compared to *wt* controls. **(B)** Expression of various VH gene segments in the repertoire of VDJ-hCµ transcripts compared to endogenous fully murine VDJ-mCµ transcripts. **(C)** Faint expression of human IgM in gut associated lymphoid tissues from LSRµKI mice, compared to a negative *wt* control and a positive control (InEps = µϵKI mice carrying a hCµ insertion immediately downstream of the *IgH* JH region and producing high amount of hIgM). **(D)** Quantitative evaluation of JH-hCµ spliced transcripts by reverse transcription and qPCR in RNA from splenocytes of LSRµKI mice stimulated *in vitro* in various conditions. (n = 3 to 16 mice, Mann and Whitney non-parametric two-tailed test; *P < 0.05; **P < 0.01, ***P < 0.001).

Since local AID recruitment at the *3’RR* supports the occurrence of local AID-mediated DNA breaks able to recombine with *Sµ* and mediate LSR in activated B cells, the expectation from the LSRµKI model was to turn LSR events into ectopic CSR to the inserted hCµ gene, thus yielding chimeric VDJ-hCµ gene transcripts. ELISA from blood indeed confirmed the presence of minute amounts of secreted human IgM in the unimmunized SOPF mice (about 50 ng/ml), which increased by 5 to 10-fold after SRBC immunization (**Figure 2A**). The inserted hCµ gene thus is expressed in LSRµKI, yields chimeric Ab molecules with human IgM determinants and its usage at this position is responsive to immune stimulation *in vivo*.

### Functional expression of the inserted hCµ gene in LSRµKI mice

In order to evaluate the expression of hCµ transcripts and check their functionality, we amplified and characterized the spliced sequences from transcripts of the human Cµ gene, using Ig repertoire RepSeq/next-generation sequencing (NGS) experiments. IgH transcripts from lymphoid tissues notably revealed functional VDJ-hCµ transcripts related to all VH subgroups and with V and J usage that did not significantly differ from that of endogenous mouse H chain transcripts (**Figure 2B**). V regions associated with hCµ included both mutated and unmutated sequences at levels roughly similar to the V sequences associated with mouse C genes from the same RNA samples (mean SHM load was 1.68% for sequences associated with mouse Cµ, 2.21% for those associated with mouse Cγ and 2.12% for those associated with hCµ).

Surprisingly, expression of the knock-in hCμ remained low *in vivo*, and we failed to identify significant levels of hIgM^+^ cells by cell cytometry since levels remained close to the background staining. Accordingly, immunofluorescence on tissue sections failed to identify cells clearly staining for human IgM though sections of the gut mucosa-associated lymphoid tissues yielded a diffuse staining. In contrast, secreted hIgM was detectable (**Figure 2C**).

Puzzled by these observations, we explored the 3’ part of hCµ transcripts for correct splicing of membrane exons. We found correct splicing of the CH exons onto the first membrane exon (M1) (**Figure S1E**). However, splicing of the M1 onto the M2 exon was not detectable, providing a likely explanation for the absence of any detectable cells with a human IgM BCR (**Figure S1F**). Sequencing of the pertinent regions in the ectopic insert confirmed intact splice sites on M1 and M2 exons on DNA (not shown).

We also quantified JH-hCµ spliced transcripts by RT-qPCR on RNA prepared from *in vitro* stimulated B cells. Increased amounts of spliced JH-hCµ transcript were detected in various conditions of B cell stimulation, either using LPS, or at a higher level with LPS+IL4 stimulation (**Figure 2D**). Thus, functionally spliced chimeric transcripts are induced upon B cell activation *in vitro*. With the aim to improve the survival of hIgM^+^ cells *in vivo*, we bred LSRµKI mice with BCL2 overexpressing mice (34) and quantified hIgM transcripts in white blood B cells 2 months after SRBC immunization: the amount of hIgM transcripts however remained similar in double mutant mice compared to LSRµKI mice. BCL2 overexpression thus did not significantly rescued B cells expressing hIgM in these conditions (**Figure S1G**).

### Mice with LSR defects show increased production of self-reactive antibodies

As mentioned above, one possibility regarding the functional significance of LSR is that the process takes place in B cells expressing self-reactive specificities. The tiny hIgM production in LSRµKI mice was not adapted to such a detailed study of Ig specificity. Rather, we chose to explore this issue in three mouse lines featuring LSR defects, which carried a partial (c*3’RR*) (27) or a complete deletion of the 3’ RR (19) or a complete deletion of *Sµ* (26).

**Figure 3:**
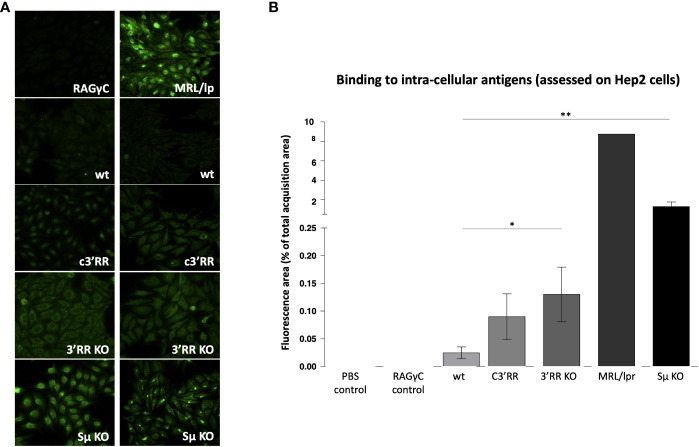
Autoantibodies against intracellular antigens detected by immunofluorescence in mice lacking LSR targeted regions. **(A)** Representative images evaluating reactivity against Hep2 cells by plasma from *wt* mice *vs* plasma from mice with either partial deletion of the *3’RR* regions targeted by LSR (c*3’RR*), complete deletion of *3’RR* (*3’RR*KO) or complete deletion of *Sµ* (*Sµ*KO). **(B)** Summary of indirect immunofluorescence detection of antibodies binding Hep2 cells, in samples from mice lacking LSR targeted regions. (n = 6 to 7 mice for wt, c3’RR, 3’KO and Sµ KO: n = 1 for the other controls, Mann and Whitney non-parametric two-tailed test; *P < 0.05; **P < 0.01).

**Figure 4:**
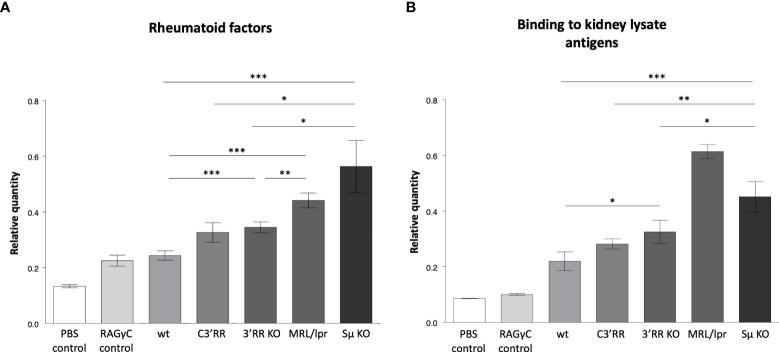
Autoantibodies detected by ELISA in mice lacking LSR targeted regions. **(A)** Rheumatoid factors by ELISA against coated rabbit immunoglobulins, in blood from *wt* mice *versus* mice with either partial deletion of the *3’RR* regions targeted by LSR (c*3’RR*), complete deletion of *3’RR* (*3’RR*KO) or complete deletion of the *Sµ* region (*Sµ*KO). **(B)** Autoantibodies against tissue antigens (from kidney lysates) in the same samples as in **(A)** (n = 2 to 13 mice, Mann and Whitney non-parametric two-tailed test; *P < 0.05; **P < 0.01, ***P < 0.001).

Using various classical assays for the detection of self-reactive Abs, blood samples from these mice readily showed increased presence of Abs binding intra-cellular Ags, the most notably in *Sµ*-deficient mice and in *3’RR* deficient mice (total or partial 3’RR deletion) to a lesser extent, as revealed by indirect immunofluorescence on Hep2 cells (**Figures 3A, B**). Presence of rheumatoid factors, again principally in *Sµ*-deleted mice (**Figure 4A**) and of Abs binding tissue antigens as demonstrated by ELISA against kidney lysate antigens (**Figure 4B**), were also readily detected. These observations suggest that regardless of the LSR target region which is deleted in the *IgH* locus, this can alter negative selection of B cells and result in increased production of self-reactive Abs.

## Discussion

AID and the *IgH 3’RR* are major players of B cell maturation by controlling SHM and CSR finally yielding high affinity BCR/Ig of different classes (2). Their most obvious action is thus to contribute to the positive selection of specific B cells in the course of immune responses. Regarding the impact of AID-mediated processes on negative selection and tolerance, several reports have documented self-reactivity together with enhanced GC reactions in AID-deficient individuals (35, 36). This was postulated to result from an SHM defect since it affects partial AID deficiencies with preserved AID-mediated recombination and CSR (36). However, a similar phenotype was also recently reported in a partial AID defect restricted to CSR while preserving SHM frequency, although this ended with lessened selection of those V segments with SHM appropriately focused on CDR segments (37). The impact of such partial AID defects on SHM *vs* CSR machinery and on B cell selection thus remains to be clarified, as well as their potential impact on the LSR process.

Evaluating a potential role for the *3’RR* in negative selection of B cells is still more difficult given its broad implication for stimulating not only AID-mediated SHM and CSR in activated B lymphocytes, but also the high-level transcription of Ig genes necessary for Ig secretion in plasma cells, as highlighted by several *3’RR* targeted disruptions in mouse models (18–20). Whether the AID-mediated loss of *IgH* C genes resulting from LSR significantly contributes to negative selection of B cells is still more elusive since it would ideally need to evaluate the BCR features and the patterns of Ig expression in cells dying from LSR.

To this aim, we designed the LSRµKI model to capture the VDJ repertoire of cells involved in LSR by introducing an “acceptor” human Cµ gene downstream of the mouse *3’RR*, to which VDJ exons recombined to the *3’RR* can then attach after transcription and splicing. As expected, this insertion of hCµ downstream of the *3’RR* had no significant effect on mature B cell compartments except for minor changes in CSR. While all previously reported alterations of the *3’RR*, such as deletions or *neo* gene insertions upstream of the most 3’ enhancer, hs4, resulted in major CSR defects, it was shown that a *neo* gene insertion downstream of hs4 and upstream of the *IgH* locus 3’ boundary had no significant effect on B cell development or CSR (38). The LSRµKI mutation thus reproduces a similar situation.

Expression of the ectopic hCµ occurred in this model but remained low, for reasons which may implicate either rare LSR events in mice, and/or premature termination of transcripts going from the VDJ to a potentially long intronic *3’RR* before reaching hCµ. Regulation of splicing is also known to involve multiple factors and may be unpredictably complex for a human gene inserted in the mouse genome at an ectopic position. In the present case, the unexpected biased splicing favoring secreted-type µ transcripts precludes expression of membrane anchored human IgM. In the absence of such a surrogate BCR to durably rescue “post-LSR” cells, the LSRµKI mice thus stand as a transient LSR reporter model.

In this reporter model, hCµ transcription and secreted human IgM evaluation indeed provide convenient read-outs for monitoring LSR events. Their analysis shows that some functional chimeric VDJ-hCµ mRNA and protein can be produced in this configuration where LSR translates into CSR towards the hCμ gene, with heightened *in vivo* production when animals are immunized, and with a repertoire of VDJ segments roughly similar to the normal B cell repertoire, not showing obvious bias in the V and J usage, and including both germline and somatically mutated VDJ regions. Although the system cannot evaluate the frequency of LSR, it indicates that the process can involve naïve as well as memory B cells. *In vitro* experiments comparing various conditions of B cell stimulations also found LSR associated with various types of stimuli.

The LSRµKI model thus provides a reporter system for LSR events, but contrary to our expectations, the hCµ inserted at this downstream position did not yield completely mature hCµ transcripts that could have supported BCR expression, and failed to support significant survival of cells “escaping LSR” by switching to an hCµ-class BCR expression. Detailed study of such cells and of their Ag specificity was thus not possible in this model.

In order to obtain an indirect evaluation of the connection between LSR and self-reactivity, we thus pursued our study by evaluating the amounts of self-reactive antibodies in mouse models where *Sµ* or *3’RR* regions targeted by LSR in the *IgH* locus, were either truncated or completely deleted. Using various classical assays for rheumatoid factors or for evaluating reactivity against tissue antigens and intra-cellular antigens, we observed that *3’RR* alterations resulted in the presence of self-reactive Abs and that higher production of such Abs was observed for the complete rather than for a partial *3’RR* deletion. Still to a higher level, self-reactive Abs were found in *Sµ*-deficient mice. Interpretation of such data can obviously be obscured by the broad consequences of the *IgH* deletions considered, with effects that are not restricted to deletion of LSR target sequences. Notably *3’RR* deletions are known for their impact on SHM (27, 39), and similarly to the hypotheses made for some AID defects focused on SHM, self-reactivity might then result from a lack of highly specific Abs, a related defect in clearing some antigens and subsequent immune dysregulation (36). However, such effects are not expected for the deletion affecting *Sµ*, from which only mechanical inhibition of CSR and LSR can be expected.

Although the data included in this report have many limitations, we think that they altogether support the hypothesis that LSR might contribute to the peripheral negative selection of self-reactive cells and thus play a role in the homeostasis of immune responses.

Alternative explanations for strong self-reactivity which develops in *Sµ*-deficient mice could also involve the CSR defect, but such a hypothesis seems unlikely, notably since other conditions associated with B cell extrinsic CSR defects, such as CD40L deficiency have not been documented as associated with increased self-reactivity. Interpretations of these deficiencies however remain controversial, as for the situation of partial AID defect, where the loss of B cell homeostasis has been principally correlated with SHM defects but also eventually with CSR defects.

Definitive conclusions about the potential role of LSR will thus await additional explorations where the ideal model would need sophisticated modifications of the locus preserving CSR, SHM and normal plasma cell differentiation, while suppressing LSR or allowing full survival of post-LSR cells (36, 37).

## Data availability statement

The data presented in the study are deposited in the GEO repository, accession number GSE233503.

## Ethics statement

The animal study was reviewed and approved by Comité pour l’éthique animale, Université de Limoges.

## Author contributions

CM designed the study. CM, LB and LNS supervised experiments. D-LN, OC, MT, BA, TO, DI, DZ, ZL, DO, C-MJ, SA, BH, CC, PS and LB contributed to the experiments. KAA provided important reagents. All authors contributed to the article and approved the submitted version.
