# Supplementary material for: Attempts to evaluate locus suicide recombination and its potential role in B cell negative selection in the mouse
Source: Front Immunol. 2023 Jun 9;14:1155906. doi: 10.3389/fimmu.2023.1155906 (PMC10288998; doi:10.3389/fimmu.2023.1155906)
Supplement: Supplementary file 1 [file DataSheet_1.pdf]

Supplemental information

Supplemental figure 1: Generation and validation of LSRµKI mice. **A**. Southern blot analysis of ES clones after neomycin selection. Genomic DNA was digested with EcoRI and hybridized with a 3' probe. **B**. Multiplex PCR to detect the *IgH wt* versus KI alleles. **C**. Gating strategy used to analyse B splenocytes by flow cytometry. **D**. Early B cell development in bone marrow. **E**. RT-PCR experiments showing efficient splicing between the CH and M1 exons of hCµ gene. **F**. RT-PCR experiment targeting the splice junction between the M1 and M2 membrane exons of the hCµ gene. **G**. hIgM expression in white blood cells of wt, LSRµKI and LSRµKI x BCL2 mice quantified by RT-qPCR. (n = 2 to 6).

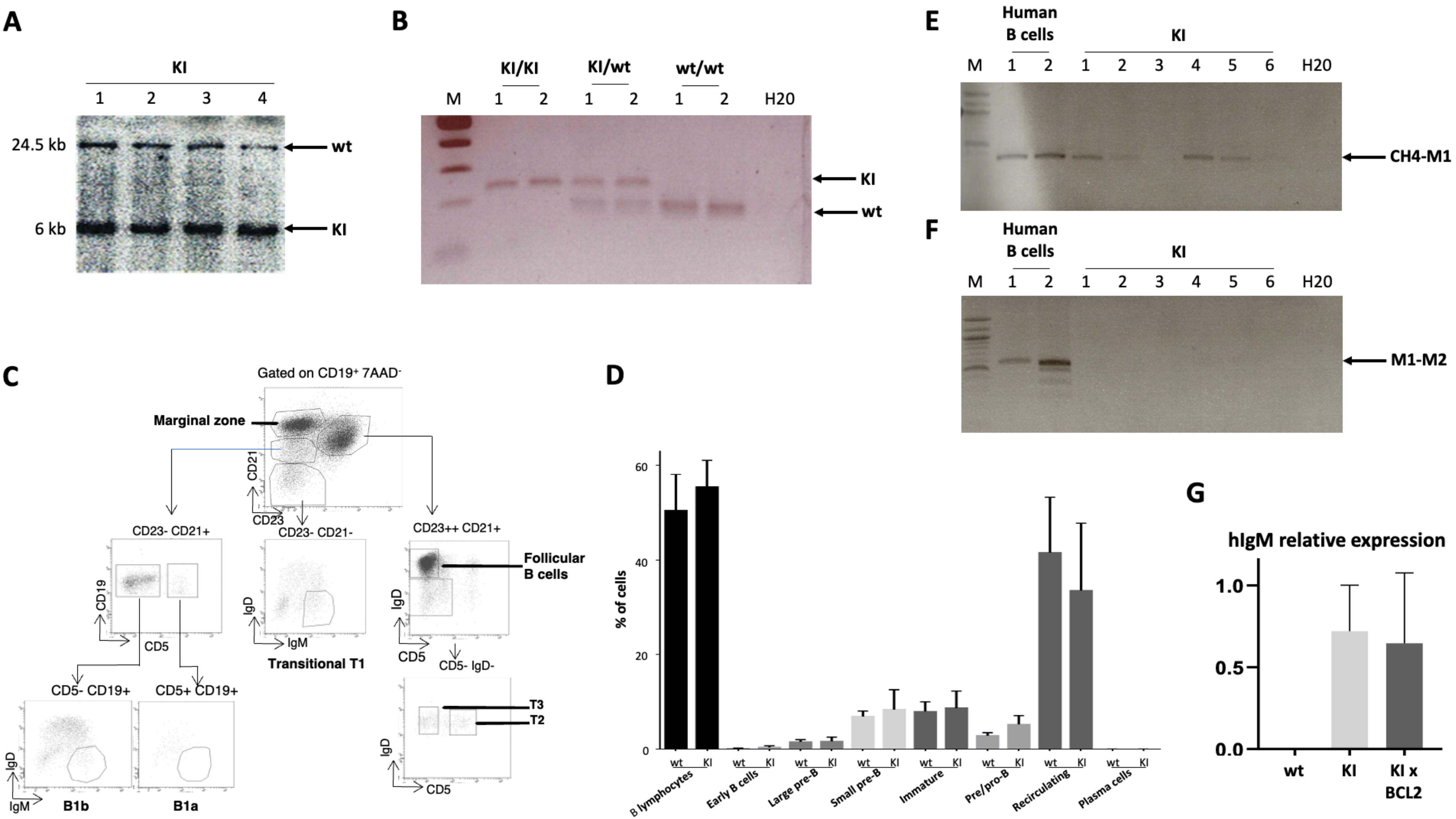

A
KI
1 2 3 4
24.5 kb
6 kb
wt
KI
B
M
KI/KI
KI/wt
wt/wt
1 2 1 2 1 2
H20
KI
wt
E
Human B cells
KI
M 1 2 1 2 3 4 5 6 H20
CH4-M1
F
Human B cells
KI
M 1 2 1 2 3 4 5 6 H20
M1-M2
C
Gated on CD19+ 7AAD-
Marginal zone
CD21
CD23
CD23- CD21+
CD23- CD21-
CD23++ CD21+
Follicular B cells
CD19
CD5
IgD
IgM
Transitional T1
CD5- IgD-
T3
T2
CD5- CD19+
CD5+ CD19+
B1b
B1a
D
% of cells
0
20
40
60
wt KI
B lymphocytes
Early B cells
Large pre-B
Small pre-B
Immature
Pre/pro-B
Recirculating
Plasma cells
G
hIgM relative expression
0.0
0.5
1.0
wt
KI
KI x BCL2

Supplementary Table 1: Primers used for repertoire amplification and sequencing.

Primer	Description	Sequence
First-strand cDNA synthesis (RACE-RepSeq)		
CAP-Race	5′ – template-switch adaptor	AAGCAGTGGTATCAACGCAGAGTACAT(GGGG) (*nucleotides in parentheses are ribonucleotides*)
Human Cmu cDNA :		
hCmu-race	Human μ H-chain cDNA synthesis primer	CGGGTRCTGCTGATGTCAGA
Mouse cDNAs :		
mmCmu-race	Mouse μ H-chain cDNA synthesis primer	CAGGTGAAGGAAATGGTGCT
mmCgamma-race	Mouse γ H-chain cDNA synthesis primer	ACAGTCACTGAGCTGCTGAG
mmCalpha-race	Mouse α H-chain cDNA synthesis primer	CGAGGGCAGGTGGAAAGTT
First PCR amplification (RACE-RepSeq)		
Long-Fw	Step-out primer 1 mix anneals on the switch	TCGTCGGCAGCGTCAGATGTGTATAAGAGACAG**CTAATACGACTCACTATAGGGCAAGCAGTGGTATCAACGCAGAGT**
Short-Fw	adaptor with Illumina (i5) adapters	TCGTCGGCAGCGTCAGATGTGTATAAGAGACAG**CTAATACGACTCACTATAGGGC**
hCmu-nested	Human μ chain nested reverse primer – (i7) adapter	GTCTCGTGGGCTCGGAGATGTGTATAAGAGACAG**CTCGTATCCGACGGGGAATT**
mmCmu-nested	Mouse μ chain nested reverse primer – (i7) adapter	GTCTCGTGGGCTCGGAGATGTGTATAAGAGACAG**GGGAAGACATTTGGGAAGGA**
mmCgamma-nested	Mouse γ chain nested reverse primer – (i7) adapter	GTCTCGTGGGCTCGGAGATGTGTATAAGAGACAG**ACTGGCTCAGGGAAATAGCC**
mmCalpha-nested	Mouse α chain nested reverse primer – (i7) adapter	GTCTCGTGGGCTCGGAGATGTGTATAAGAGACAG**TCAGGCAGCCGATTATCACTGGG**
Second PCR amplification		
Illu Fw 1	Illumina Index 2 (i5=S513) adapters	AATGATACGGCGACCACCGAGATCTACACTCGACTAGTCGTCGGCAGCGTC
Illu Fw 2	Illumina Index 2 (i5=S515) adapters	AATGATACGGCGACCACCGAGATCTACACTTCTAGCTTCGTCGGCAGCGTC
Illu Fw 3	Illumina Index 2 (i5=S516) adapters	AATGATACGGCGACCACCGAGATCTACACCCTAGAGTTCGTCGGCAGCGTC
Illu Fw 4	Illumina Index 2 (i5=S517) adapters	AATGATACGGCGACCACCGAGATCTACACGCGTAAGATCGTCGGCAGCGTC
Illu Fw 5	Illumina Index 2 (i5=S518) adapters	AATGATACGGCGACCACCGAGATCTACACCTATTAAGTCGTCGGCAGCGTC
Illu Fw 6	Illumina Index 2 (i5=S520) adapters	AATGATACGGCGACCACCGAGATCTACACAAGGCTATTCGTCGGCAGCGTC
Illu Fw 7	Illumina Index 2 (i5=S521) adapters	AATGATACGGCGACCACCGAGATCTACACGAGCCTTATCGTCGGCAGCGTC
Illu Fw 8	Illumina Index 2 (i5=S522) adapters	AATGATACGGCGACCACCGAGATCTACACTTATGCGATCGTCGGCAGCGTC
Illu Rev 1	Illumina Index 1 (i7=N716) adapters	CAAGCAGAAGACGGCATACGAGATTAGCGAGTGTCTCGTGGGCTCGG
Illu Rev 2	Illumina Index 1 (i7=N718) adapters	CAAGCAGAAGACGGCATACGAGATGTAGCTCCGTCTCGTGGGCTCGG
Illu Rev 3	Illumina Index 1 (i7=N719) adapters	CAAGCAGAAGACGGCATACGAGATTACTACGCGTCTCGTGGGCTCGG
Illu Rev 4	Illumina Index 1 (i7=N720) adapters	CAAGCAGAAGACGGCATACGAGATAGGCTCCGGTCTCGTGGGCTCGG
Illu Rev 5	Illumina Index 1 (i7=N721) adapters	CAAGCAGAAGACGGCATACGAGATGCAGCGTAGTCTCGTGGGCTCGG
Illu Rev 6	Illumina Index 1 (i7=N722) adapters	CAAGCAGAAGACGGCATACGAGATCTGCGCATGTCTCGTGGGCTCGG
Illu Rev 7	Illumina Index 1 (i7=N723) adapters	CAAGCAGAAGACGGCATACGAGATGAGCGCTAGTCTCGTGGGCTCGG
Illu Rev 8	Illumina Index 1 (i7=N724) adapters	CAAGCAGAAGACGGCATACGAGATCGCTCAGTGTCTCGTGGGCTCGG
